# Prediction of Reactivation After Antivascular Endothelial Growth Factor Monotherapy for Retinopathy of Prematurity: Multimodal Machine Learning Model Study

**DOI:** 10.2196/60367

**Published:** 2025-04-23

**Authors:** Rong Wu, Yu Zhang, Peijie Huang, Yiying Xie, Jianxun Wang, Shuangyong Wang, Qiuxia Lin, Yichen Bai, Songfu Feng, Nian Cai, Xiaohe Lu

**Affiliations:** 1 Department of Ophthalmology Zhujiang Hospital Southern Medical University Guangzhou China; 2 School of Information Engineering Guangdong University of Technology Guangzhou China; 3 Department of Pediatric Ophthalmology Guangzhou Children’s Hospital and Guangzhou Women and Children’s Medical Center Guangzhou Medical University Guangzhou China; 4 Department of Ophthalmology Third Affiliated Hospital of Guangzhou Medical University Guangzhou China

**Keywords:** retinopathy of prematurity, reactivation, prediction, machine learning, deep learning, anti-VEGF

## Abstract

**Background:**

Retinopathy of prematurity (ROP) is the leading preventable cause of childhood blindness. A timely intravitreal injection of antivascular endothelial growth factor (anti-VEGF) is required to prevent retinal detachment with consequent vision impairment and loss. However, anti-VEGF has been reported to be associated with ROP reactivation. Therefore, an accurate prediction of reactivation after treatment is urgently needed.

**Objective:**

To develop and validate prediction models for reactivation after anti-VEGF intravitreal injection in infants with ROP using multimodal machine learning algorithms.

**Methods:**

Infants with ROP undergoing anti-VEGF treatment were recruited from 3 hospitals, and conventional machine learning, deep learning, and fusion models were constructed. The areas under the curve (AUCs), accuracy, sensitivity, and specificity were used to show the performances of the prediction models.

**Results:**

A total of 239 cases with anti-VEGF treatment were recruited, including 90 (37.66%) with reactivation and 149 (62.34%) nonreactivation cases. The AUCs for the conventional machine learning model were 0.806 and 0.805 in the internal validation and test groups, respectively. The average AUC, sensitivity, and specificity in the test for the deep learning model were 0.787, 0.800, and 0.570, respectively. The specificity, AUC, and sensitivity for the fusion model were 0.686, 0.822, and 0.800 in a test, separately.

**Conclusions:**

We constructed 3 prediction models for ROP reactivation. The fusion model achieved the best performance. Using this prediction model, we could optimize strategies for treating ROP in infants and develop better screening plans after treatment.

## Introduction

Retinopathy of prematurity (ROP) is characterized by retinal ischemia-hypoxia in preterm infants. Worldwide, it is a leading cause of vision loss and blindness in childhood [1-3]. More than 20,000 infants experience blindness, and an estimated 12,300 infants have different levels of visual impairment due to ROP [4]. A dysregulation of vascular endothelial growth factor (VEGF) has been proven to play an important role in the development of ROP [5,6]. Laser photocoagulation and anti-VEGF agents are the mainstay treatments for ROP. The clinical application of intravitreal injection (IVI) with anti-VEGF agents has recently increased due to their fewer side effects and more advantages, including a lower risk of future myopia, better peripheral vision, and faster regression of acute-phase ROP [7-10]. However, ROP reactivation after anti-VEGF therapy is still a concern. Given the short half-life of these agents, the beneficial effects of anti-VEGF might be transient, potentially increasing the risk of ROP reactivation. Previous studies evaluating the clinical outcomes of anti-VEGF agents for ROP have reported reactivation rates between 6.8% and 64% after treatment [8,9,11-13].

Early detection and timely treatment upon reactivation is critical for infants with ROP who undergo anti-VEGF therapy. Previous studies on ROP reactivation after anti-VEGF therapy have mainly focused on the risk factors of reactivation. Factors related to ocular conditions include zone I ROP, severe retinal neovascularization, preretinal hemorrhage, and aggressive ROP [12,14]. Neonatal factors include low gestational age, low birth weight, early postmenstrual age (PMA) at initial treatment, and low Apgar scores [12]. Maternal factors include multiple births [12]. Other risk factors related to neonatal interventions include oxygen requirement before or after treatment and a longer duration of hospitalization [9]. Prediction models that can identify infants with a high risk of ROP reactivation are needed in clinical practice.

Artificial intelligence has recently optimized medical practice [15-17]. Artificial intelligence has been mainly applied to ROP diagnosis and prediction based on imaging [17-19]. To our knowledge, studies on ROP reactivation after treatment are very limited, and there is no successful prediction model for clinical application. Machine learning is a subset of artificial intelligence and includes conventional machine learning and deep learning [20-22]. In this study, we developed prediction models for reactivation after anti-VEGF treatment in infants with ROP using machine learning algorithms based on clinical risk factors and retinal images before treatment.

## Methods

### Ethical Considerations

This study was approved by the study hospitals’ institutional ethics committees (2022-KY-143) and adhered to the tenets of the Declaration of Helsinki. All parents or guardians of the recruited infants provided written informed consent prior to participation. Data were anonymized and deidentified before analysis.

### Study Population

We collected data retrospectively on infants who received anti-VEGF monotherapy for ROP requiring treatment between April 2016 and November 2022 at Hospital 1 (Zhujiang Hospital, Southern Medical University), Hospital 2 (The Third Affiliated Hospital, Guangzhou Medical University), and Hospital 3 (Guangzhou Children’s Hospital and Guangzhou Women and Children’s Medical Center), all in Guangzhou, China. Infants with incomplete data or any other ocular diseases besides ROP were excluded. Additionally, infants who underwent anti-VEGF therapy as adjunctive treatment before planned vitrectomy or received follow-up examinations for less than 12 months were excluded.

### Ocular Examinations

During each ROP screening examination, retinal photographs were captured using the RetCam Ⅲ digital fundus camera (Natus). The diagnosis of ROP and the indication for its treatment were based on the International Classification of ROP Revisited and the Early Treatment for ROP study, respectively. Treatment-requiring ROP included threshold disease, stage 4 or 5 ROP, and type 1 or aggressive ROP. Ocular examinations were conducted before and on days 1, 7, 14, and 28 after anti-VEGF therapy, and either biweekly or monthly depending on the ocular findings and systemic conditions. A reactivation of ROP was defined as the recurrence of acute phase features including a range of signs from a new demarcation line to reactivated stage 3 with disease, vascular dilation, tortuosity, or new/recurrent neovascularization that required further treatment [13].

### IVI of Anti-VEGF

All parents or guardians of the infants were fully informed of the efficacy and possible complications prior to IVI of conbercept, and they provided written informed consent. The anti-VEGF treatment was performed as monotherapy for treatment-naive patients. Anti-VEGF agents were injected intravitreally at 1.5 mm posterior to the limbus with a 30-gauge needle under topical aesthesia. Topical tobramycin dexamethasone was administered for 3 days after the injection. All operations were done by a trained pediatric ophthalmologist (author SF).

### Clinical Risk Factors

Based on previous studies and clinical experience, the potential clinical risk factors of ROP reactivation extracted from electronic medical records included maternal factors, neonatal factors, ocular conditions, laboratory factors, and neonatal interventions. Specifically, maternal factors of interest included maternal age, gestational hypertension, gestational diabetes mellitus, premature rupture of membranes, cesarean delivery, and in vitro fertilization and embryo transfer. Neonatal factors included gestational age, birth weight, PMA at initial ROP treatment, fetal distress, sex, small for gestational age, Apgar scores (1 and 5 min), multiple births, asphyxia, sepsis, respiratory distress syndrome, bronchopulmonary dysplasia, pneumonia, intraventricular hemorrhage, necrotizing enterocolitis, hypoxic-ischemic encephalopathy, atrial septal defect, patent foramen ovale, patent ductus arteriosus, and hyperbilirubinemia. Ocular conditions included zone I ROP, preretinal hemorrhage, and aggressive ROP. The hemoglobin concentration before treatment was included among the laboratory factors. Finally, neonatal interventions included mechanical ventilation and oxygen therapy (before treatment) [9,11,12].

In the data processing stage, any missing data were handled according to the type of variable. For continuous data, we imputed missing values by calculating the mean within each category based on the label (0 or 1). For discrete data, we used the mode within each category to fill in missing values. This approach ensured that the values imputed were representative of their respective categories, thus minimizing bias in the analysis.

### Image Collection and Case Labeling

All retinal photographs were captured using the commercial RetCam camera. Retinal photographs of poor photographic quality were excluded by 2 experienced ophthalmologists (Authors SF and RW). Since the prediction for ROP reactivation would be performed independently on each infant rather than each retinal photograph, retinal photographs of both eyes from the same infant were labeled as a single case. All cases were labeled independently by 2 clinical ophthalmologists (SF and RW). Based on the ocular findings after anti-VEGF therapy, each case was annotated reactivation or nonreactivation. If ROP reactivation occurred in one of the eyes, the case would be labeled as reactivation.

The κ was 0.81 for annotation of ROP reactivation, indicating good agreement between the 2 ophthalmologists in labeling. Moreover, the labels were further confirmed by a senior retinal specialist (author XL) to generate a final annotation. These annotations were used as ground-truth labels in the development and validation of the prediction models for ROP reactivation.

### Development and Validation of the Prediction Models

#### Conventional Machine Learning Model

**Figure 1:**
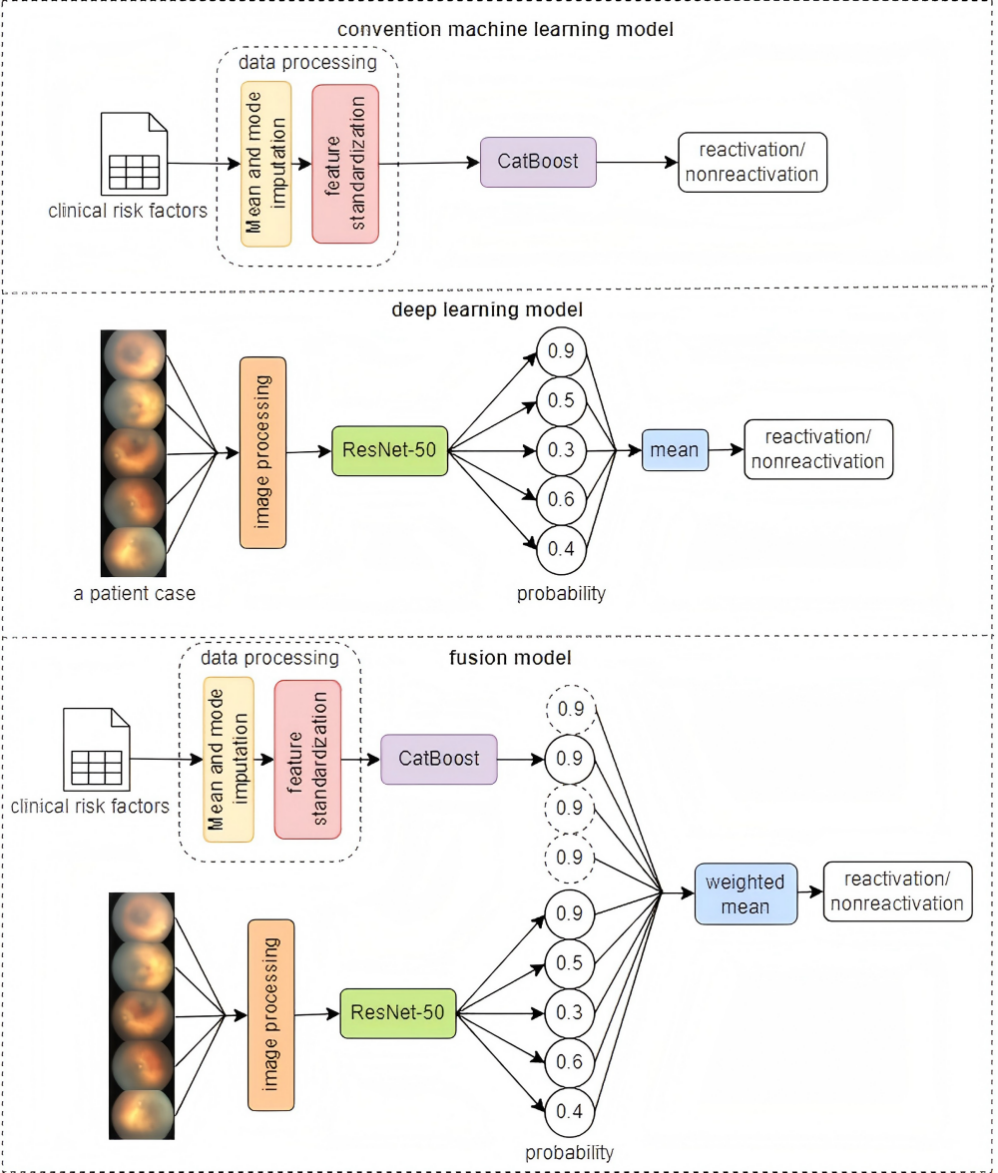
Illustration of the proposed algorithmic pipeline. CatBoost: Categorical Boosting.

An illustration of the conventional machine learning prediction model is presented in Figure 1. The importance ranking of the clinical risk factors for ROP reactivation was assessed, and prediction models based on clinical risk factors were developed using conventional machine learning algorithms. The process was implemented with Python (version 3.9; Python Software Foundation).

During the data preprocessing stage, we employed 2 filling strategies. For discrete data, we used mean filling followed by rounding; for continuous data, we used mean filling. Subsequently, the continuous data underwent standardization, transforming the data into a standard normal distribution with a mean of 0 (SD 1), ensuring uniform scales across different features. This helped mitigate potential model biases arising from scale differences, enhancing overall model robustness and performance.

In this study, we employed a comprehensive approach to assess the feature importance in predictive models, utilizing 5 different algorithms: random forest (RF), Adaptive Boosting (AdaBoost), Extreme Gradient Boosting (XGBoost), Categorical Boosting (CatBoost), and logistic regression (LR). Table S1 in Multimedia Appendix 1 contains the parameter details. The method involves ranking the feature importance or weights for each algorithm, whereas for LR, feature importance was determined by the absolute values of the weights. Subsequently, these rankings were visualized using scatter plots, with each feature represented along the x-axis and its importance rank along the y-axis. This comprehensive visualization offered a comparative analysis of feature contributions across different algorithms, providing insights into the robustness and consistency of the feature importance.

We conducted an in-depth investigation into the impact of varying the number of selected features (N) on the predictive model performance, employing 5 distinct algorithms. We systematically varied the number of selected features, choosing from the set (5, 10, 15, 20, 25, and 30), and assessed the resulting model performance by calculating the area under the curve (AUC) for each model across different feature subsets.

We conducted a comprehensive evaluation of the 5 different predictive models, with a specific focus on their receiver operating characteristic (ROC) curves. The models were trained and evaluated using all features on a standardized data set. The emphasis of this research lies in presenting the performance of different models through ROC curves, offering crucial insights into their predictive capabilities. Furthermore, we conducted 10 iterations of training for each of the 5 distinct predictive models and computed their average AUC values on the corresponding validation sets, along with their respective SDs.

#### Deep Learning Model

Figure 1 presents an illustration of the deep learning prediction model. Retinal photographs captured before anti-VEGF therapy of infants with ROP were obtained to develop a prediction model using deep learning algorithms. ResNet-50, a deep convolutional neural network classifier pretrained on the ImageNet database, was fine-tuned with our data set to predict ROP reactivation. The process was implemented with Python (version 3.9) and PyTorch (version 1.11.0).

We designed a series of image preprocessing methods beginning with feature enhancement and noise suppression using median filtering. This technique was chosen for its effectiveness in removing isolated noise pixels while maintaining the clarity of important features such as blood vessel edges. Subsequently, the images were converted to grayscale for processing to reduce computational complexity.

For the training set, we employed a series of preprocessing steps. These included resizing the images to 224 × 224 pixels, introducing RandAugment for random data augmentation, and applying both random horizontal and vertical flips. These measures aimed to increase the diversity of the data, enhancing the model's robustness. For the internal validation and test sets, we consistently resized the images to 224 × 224 pixels and transformed them into tensors. This ensured uniformity in the model's input during the validation process. This diversified preprocessing approach helped the model adapt to the distinct characteristics of each data set, ultimately improving its generalization performance.

We employed a pretrained ResNet-50 model and iteratively trained it on the image training set, incorporating image preprocessing and data augmentation techniques to enhance the model's generalization performance. We adopted the cross-entropy loss function, whereby the model learned to adjust its parameters by minimizing the cross-entropy loss, enabling it to more accurately predict the samples’ categories. During the training process, we utilized the Adam optimizer and a cosine annealing learning rate scheduler, conducting a total of 100 epochs. At the end of each epoch, we evaluated the model's performance on both an internal validation set and an external test set. Throughout the training, we also applied the Exponential Moving Average (EMA) technique to stabilize the model parameters and improve the overall model generalization.

We constructed a data set in which each case consisted of multiple medical images and randomly selected 5 images per patient for the experiment. When performing case-level predictions, we treated the model's output as the probability score for the entire case. By averaging the probability scores from multiple images within each case, we computed the final case-level prediction results.

To enhance the visualization and interpretability of the ROP reactivation prediction, we applied the Gradient-Weighted Class Activation Mapping (Grad-CAM) technique to generate heat maps of key regions. This technique highlights areas crucial to ROP reactivation prediction by analyzing feature weights in specific network layers, such as layer 4 of ResNet50. This approach allows us to intuitively identify retinal features that significantly contribute to the prediction outcome.

#### Fusion Model

An illustration of the prediction model combining the conventional machine learning model and the deep learning model is presented in Figure 1. Among the 5 conventional machine learning prediction models, the model based on the CatBoost algorithm exhibited the best predictive performance. We weighed and averaged the probability outputs of the optimal conventional machine learning model and the deep learning model to obtain the final prediction result. The process was implemented with Python (version 3.9) and PyTorch (version 1.11.0).

First, we modeled the basic clinical features of patients using a risk factor model, considering traditional risk factors such as maternal age and infant weight. Subsequently, we employed a deep learning model (ResNet-50) to process retinal images of newborns, capturing more comprehensive information. The deep learning model, by learning features from images, can capture complex patterns that traditional models find challenging.

To fully leverage the strengths of both models, we treated the 5 probability scores generated by the deep learning model for each image as equal in weight and assigned the output of the risk factor model a weight equivalent to that of 4 images, thereby producing a more accurate and robust prediction result. We obtained the deep learning probability scores for each sample through independent training on the training set, simultaneously predicting risk factor probabilities using the risk factor model. Finally, we linearly combined the probability scores from both models to derive the ultimate fusion result.

### Statistical Analysis

Statistical analyses were performed using R software (version 3.0.2; R Core Team). Categorical variables were expressed as numbers and percentages and analyzed using chi-square tests. If any cell number was less than 5, the Fisher exact test was applied. Continuous variables conforming to normal distribution were expressed as mean and SD and compared using the independent 2-sample *t* test. If a normal distribution was not confirmed, the median and interquartile ranges were used, and the Mann-Whitney U test was performed. Breakdowns of the predictive labels with reference to the ground-truth labels were depicted as confusion matrices, which were used to calculate the accuracy, sensitivity, and specificity of the prediction models with the 3 training schemes using Python (version 3.7.0). *P*＜.05 was considered statistically significant.

## Results

### Demographics of the Study Groups

**Figure 2:**
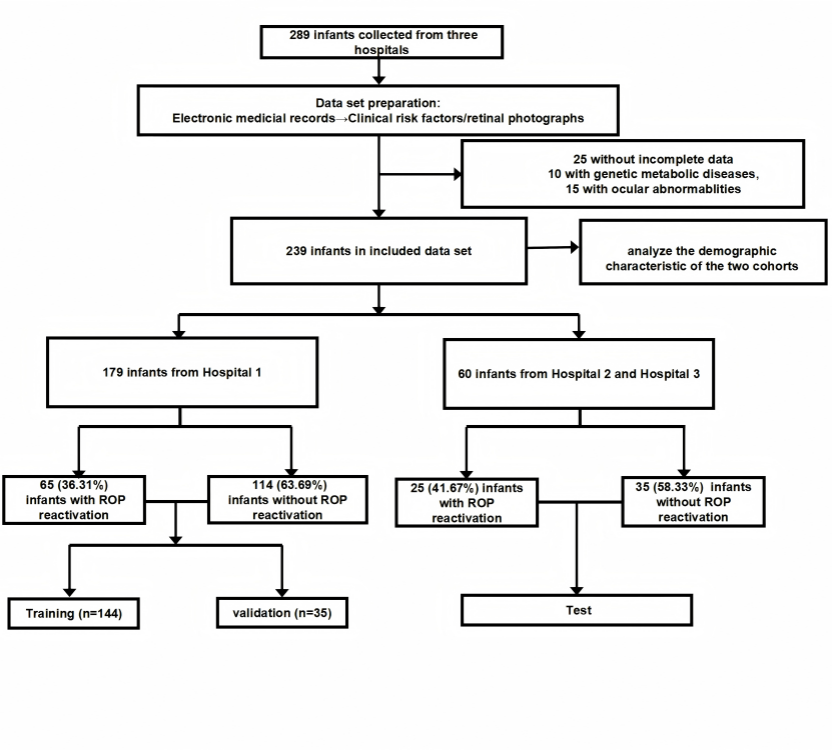
The flow chart of patient cohorts. ROP: retinopathy of prematurity.

The study group finally included 239 infants. The infant recruitment flow is presented in Figure 2. The detailed demographic characteristics of the enrolled infants are summarized in Table 1. Among the 179 included infants of Hospital 1, the mean gestational age was 28.87 (SD 2.46) weeks, and the mean birth weight was 1.18 (SD 0.35) kg. Among the 60 infants enrolled from Hospital 2 and Hospital 3, the mean gestational age was 28.30 (SD 1.90) weeks, and the mean birth weight was 1.09 (SD 0.28) kg. There was no significant difference between the demographic characteristics of the 2 cohorts (all *P*>.05).

**Table 1:** Demographic characteristics of all the enrolled infants.

Variables		All infants (N=239)	Training (n=179)	Test (n=60)	*P* value
**Maternal factors**					
	Maternal age (years), mean (SD)	30.72 (5.32)	30.65 (5.08)	30.95 (6.03)	.73
	In vitro fertilization and embryo transfer, n (%)	41 (17.15)	31 (17.32)	10 (16.67)	.91
	Gestational hypertension, n (%)	30 (12.55)	24(13.41)	6 (10)	.49
	Gestational diabetes mellitus, n (%)	34 (14.23)	25 (13.97)	9 (15)	.84
	Cesarean delivery, n (%)	105 (43.93)	79 (44.13)	26 (43.34)	.91
	Premature rupture of membranes, n (%)	37 (15.48)	26 (14.53)	11 (17.74)	.48
**Neonatal factors**					
	Gestational age (weeks), mean (SD)	28.66 (2.38)	28.87 (2.46)	28.30 (1.90)	.07
	Birth weight (kg), mean (SD)	1.15 (0.34)	1.18 (0.35	1.09 (0.28)	.05
	Multiple gestations, n (%)	58 (24.27)	46 (25.70)	12 (20)	.37
	Small for gestational age, n (%)	39 (16.32)	30 (16.76)	9 (15)	.75
	Intrauterine distress, n (%)	22 (9.20)	18 (10.06)	4 (6.67)	.43
	Postmenstrual age at initial ROP^a^ treatment (weeks), mean (SD)	36.31 (2.29)	36.29 (2.31)	36.38 (2.25)	.78
	Male sex, n (%)	131 (54.81)	94 (52.51)	37 (61.67)	.22
	Apgar scores at 1 minute, mean (SD)	7.35 (2.05)	7.38 (2.07)	7.25 (2.01)	.67
	Apgar scores at 5 minutes, mean (SD)	8.58 (1.39)	8.62 (1.43)	8.58 (1.29)	.98
	Asphyxia, n (%)	98 (41)	72 (40.2)	26 (43.33)	.67
	Respiratory distress syndrome, n (%)	179 (75.24)	130 (72.63)	49 (81.67)	.16
	Bronchopulmonary dysplasia, n (%)	146 (61.09)	107 (59.78)	39 (65)	.47
	Sepsis, n (%)	65 (27.20)	47 (26.26)	18 (30)	.57
	Necrotizing enterocolitis, n (%)	37 (15.48)	27 (15.08)	10 (16.67)	.55
	Intraventricular hemorrhage, n (%)	107 (44.76)	80 (44.69)	27 (45)	.97
	Hypoxic-ischemic encephalopathy, n (%)	48 (20.08)	35 (19.55)	13 (21.67)	.72
	Patent ductus arteriosus, n (%)	90 (37.66)	70 (39.11)	20 (33.33)	.42
	Atrial septal defect, n (%)	39 (16.32)	27 (15.08)	12 (20)	.37
	Patent foramen ovale, n (%)	183 (76.57)	139 (77.65)	44 (73.33)	.49
	Hyperbilirubinemia, n (%)	148 (61.92)	110 (61.45)	38 (63.33)	.80
	Pneumonia, n (%)	140 (58.58)	103 (57.54)	37 (61.67)	.58
	Hemoglobin concentration (g/L), mean (SD)	125.04 (26.67)	124.85 (25.78)	125.60 (29.38)	.85
	Mechanical ventilation, n (%)	214 (89.54)	160 (89.39)	54 (90)	.89
	Oxygen therapy (before treatment), n (%)	234 (97.91)	175 (97.77)	58 (96.67)	.64
**Ocular conditions**					
	Zone 1 ROP, n (%)	65 (27.20)	44 (24.58)	21 (35)	.12
	Aggressive ROP, n (%)	40 (16.74)	31 (17.32)	9 (15)	.68
	Retinal hemorrhage, n (%)	71(29.71)	49(27.37)	22(36.67)	.17

^a^ROP: retinopathy of prematurity.

### Predictive Performance of the Conventional Machine Learning Models

Using all clinical risk factors, the ROC curves and AUC values of the models with different algorithms are compared in Figure S1 in Multimedia Appendix 1. CatBoost had the best performance, with a mean AUC of 0.812 (SD 0.012), as shown in Table 2. Through comprehensive visualization offered by a comparative analysis of feature contributions across different algorithms, the top 20 features were ranked. The top 5 predictors were gestational age, birth weight, PMA at the initial IVI treatment, pneumonia, and hemoglobin concentration (Figure S2 in Multimedia Appendix 1).

**Table 2:** Predictive performances of the 5 models.

Algorithm	AUC^a^, mean (SD)
AdaBoost	0.808 (0.008)
Random Forest	0.803 (0.020)
XGBoost^b^	0.780 (0.005)
CatBoost^c^	0.812 (0.012)
Logistic regression	0.720 (0.005)

^a^AUC: area under the curve.

^b^XGBoost: Extreme Gradient Boosting.

^c^CatBoost: Categorical Boosting.

**Figure 3:**
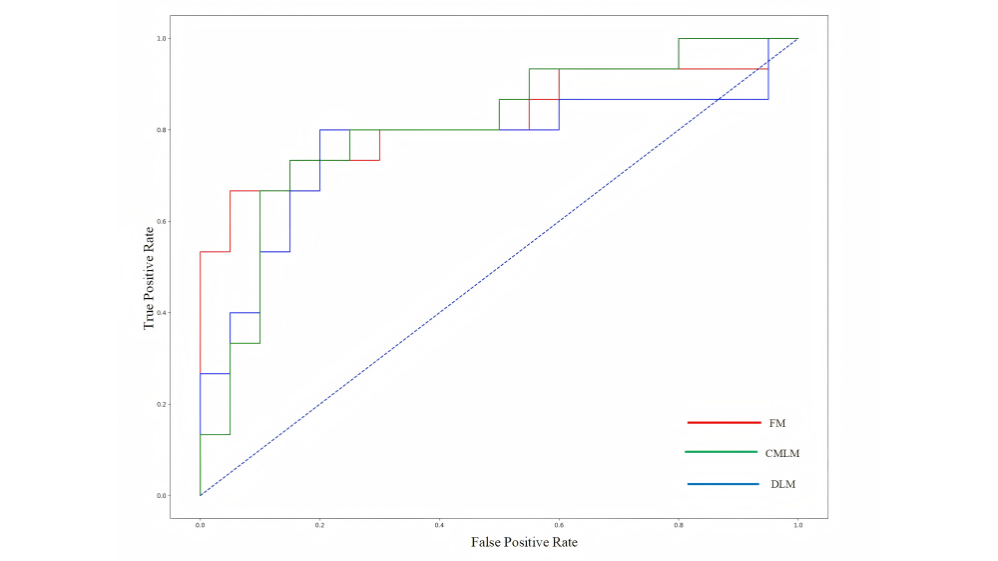
Receiver operating characteristic (ROC) curves of 3 models in the internal validation cohort. CMLM: conventional machine learning model; DLM: deep learning model; FM: fusion model.

**Figure 4:**
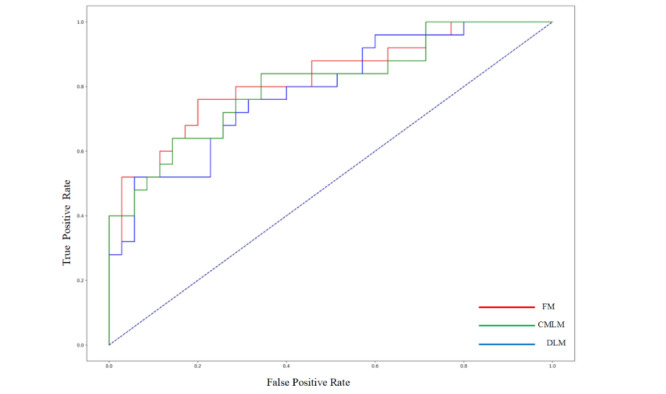
Receiver operating characteristic (ROC) curves of 3 models in the test cohort. CMLM: conventional machine learning model; DLM: deep learning model; FM: fusion model.

To evaluate the relationship between the number of variables and predictive performance, the top 5, 10, 15, 20, 25, and 30 variables were introduced to 5 algorithms successively. The performance of most models plateaued when 20 variables were introduced. When more than 20 variables were introduced, the performance of CatBoost continued to improve slightly with the addition of variables, while the performance of RF and LR began to deteriorate (Figure S3 in Multimedia Appendix 1). Finally, the CatBoost prediction model with the top 20 variables had the best performance. The AUCs, sensitivities, and specificities of the CatBoost prediction model were 0.806, 0.800, and 0.750 for the internal validation, respectively, and 0.805, 0.800, and 0.657 for the test, respectively (Table 3 and Figures 3 and 4). The confusion matrix is presented in Figure S4 in Multimedia Appendix 1.

**Table 3:** Performance comparison of the different models.

Model	ACC^a^	SEN^b^	SPE^c^	AUC^d^
CMLM^e^_ val^f^	0.771	0.800	0.750	0.806
CMLM_ test	0.716	0.800	0.657	0.805
DLM^g^_ val	0.771	0.733	0.800	0.767
DLM_ test	0.667	0.800	0.570	0.787
FM^h^_ val	0.742	0.800	0.700	0.823
FM_ test	0.733	0.800	0.686	0.822

^a^ACC: accuracy.

^b^SEN: sensitivity.

^c^SPE: specificity.

^d^AUC: area under the curve.

^e^CMLM: conventional machine learning model.

^f^Val: internal validation.

^g^DLM: deep learning model.

^h^FM: fusion model.

### Predictive Performance of the Deep Learning Model

**Figure 5:**
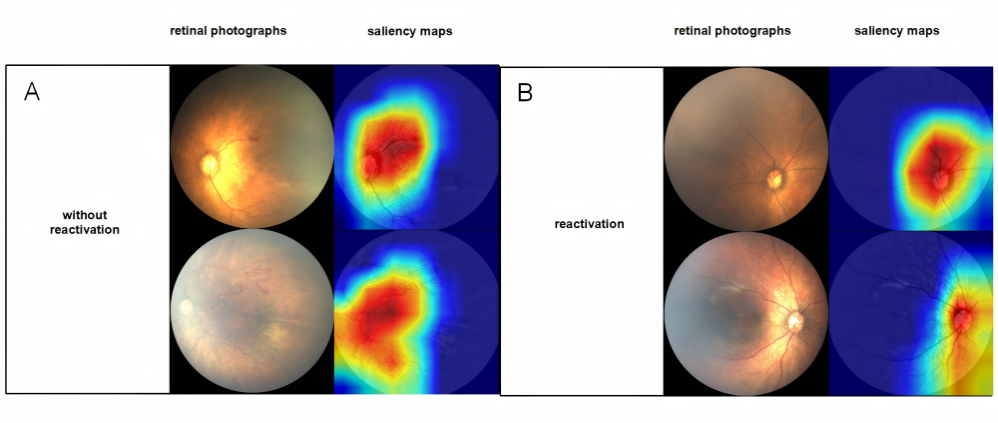
Original retinal photographs and saliency maps of reactivation and without reactivation cases from Grad-CAM.

The predictive performances of the deep learning model based on retinal photographs captured before anti-VEGF therapy in internal validation and test are summarized in Table 3 and Figures 3 and 4. The AUCs, sensitivities, and specificities of the deep learning prediction model were 0.767, 0.733, and 0.800 for the internal validation, respectively, and 0.787, 0.800, and 0.570 for the test, respectively. To improve the interpretability of the model, we used Grad-CAM to visualize the key regions in retinal photographs highly associated with ROP reactivation. Several representative examples of retinal photographs with accompanying saliency maps are shown in Figure 5. The optic and the retinal vessels were mainly used to predict reactivation. The confusion matrix is presented in Figure S5 in Multimedia Appendix 1.

### Predictive Performance of the Fusion Model

Since the CatBoost model had the best predictive performance among the 5 conventional machine learning models, it was deployed to be combined with the deep learning model. The AUCs of the fusion prediction model were 0.823 for the internal validation and 0.822 for the test. The confusion matrix is presented in Figure S6 in Multimedia Appendix 1.

## Discussion

### Principal Findings

Given the risk of ROP reactivation after anti-VEGF therapy and the crucial need for timely intervention, this study established prediction models for ROP reactivation after anti-VEGF treatment using machine learning algorithms based on pretreatment data, including clinical risk factors and retinal images. The machine learning prediction models achieved promising performance and were externally validated in a group of infants from different hospitals.

The ROP reactivation prediction models can optimize ophthalmologists’ clinical decision-making before anti-VEGF therapy. Fluorescein angiography under anesthesia and, if necessary, laser treatment for residual areas of nonperfusion have been recommended for infants receiving anti-VEGF treatment who are at high risk of reactivation [23,24]. In addition, more frequent retinal examinations are needed after anti-VEGF treatment. Meanwhile, a small number of infants predicted to be at low risk of ROP reactivation experienced reactivation, and treatment was repeated in this study. Since it is not recommended to miss even one case of ROP reactivation due to its devastating visual consequences, we do not recommend discontinuing regular follow-up examinations after treating infants at low risk of ROP reactivation.

We employed 5 different algorithms to select the machine learning model with the best predictive performance. Most of them achieved high performance, among which the CatBoost model exhibited optimal performance in the internal validation and test. CatBoost is a modification of the gradient-boosted decision tree algorithm, which is compatible with categorical features [25]. In previous studies, the CatBoost algorithm has shown satisfactory performance in predicting malaria, Parkinson disease, perioperative major adverse cardiovascular events, and in-hospital mortality [26-29]. In our study, the CatBoost algorithm was combined with the greedy algorithm to identify the most predictive combination of clinical risk factors for ROP reactivation. The greedy algorithm is an algorithmic paradigm that follows a problem-solving approach to realize the locally optimal choice at each stage. PMA at the initial IVI treatment was significantly different between the non-ROP reactivation and ROP reactivation groups in this study (non-ROP reactivation: mean 36.93, SD 2.10 weeks versus ROP reactivation: mean 35.15, SD 2.21 weeks; *P*<.05). Similarly, Lyu et al [9] found that early PMA at the initial IVI treatment was a significant independent risk factor for ROP reactivation [12]. Infants with earlier PMA at initial treatment may experience worse systemic conditions and severe ROP that necessitate timely treatment. The presence of pneumonia implies more severe systemic hypoxia that can exacerbate ocular hypoxia and increase the risk of neovascularization [12]. Longer oxygen treatment for pneumonia may also increase the risk of ROP reactivation after treatment with IVI.

Recently, deep learning technology has been applied to accurately diagnose and predict ROP and its severity based on retinal photographs [18,19,30-32]. We previously described the use of a deep learning system to predict ROP and its severity before 45 weeks PMA [19]. Retinal status is important for predicting ROP progression and reactivation. Previous studies have demonstrated that early vascular dilation and tortuosity are insufficient to predict ROP. In addition, the extent of temporal retinal blood vessel immaturity at the first screening has a prognostic significance in the early course of ROP [19]. The ROP vascular severity score derived from a deep learning classifier based on retinal photographs captured within 4 weeks before and after treatment and at the time of treatment could consistently reflect disease progression and posttreatment regression of ROP. The ROP vascular severity score at the time of initial treatment and progression rate after initial screening were associated with ROP reactivation, indicating that the features of retinal photographs before initial treatment may predict ROP reactivation [10]. Although the ROP vascular severity score can help monitor ROP over time, without guidance on optimal cut points, clinicians might not be able to use the vascular severity score for ROP management [10]. In this study, retinal photographs captured before the initial anti-VEGF therapy were analyzed using deep learning algorithms and showed promising predictive value for ROP reactivation. The optic disc and the areas around the optic disc and retinal vessels may be the potential regions for ROP reactivation,

Furthermore, we integrated the prediction model based on retinal photographs and the optimal model based on clinical risk factors. The fusion model achieved the best performance for predicting ROP reactivation, suggesting that clinical information and biomarkers from retinal photographs play important roles in ROP reactivation modeling. With the development of artificial intelligence, more studies are combining image features with clinical information. Coyner et al [33] successfully improved the specificity of the ROP prediction model by adding retinal photograph features. The model combining gestational age with vascular severity score was the best-performing model. The breast cancer recurrence model using hematoxylin–eosin-stained images and clinical information accurately assessed the risk of recurrence [34]. Eilts et al [10] reported that retinal photographs before and after treatment were predictive for ROP reactivation after ranibizumab injection. Their study evaluated the potential role of an artificial intelligence–derived screening method in predicting ROP reactivation after anti-VEGF treatment. However, their study had some limitations, the main one being the small sample size, as only 19 infants were included. Moreover, only posterior retinal photographs captured from RetCam were analyzed, potentially missing the value from peripheral retinal photographs. On the other hand, in this study, we collected more cases and captured retinal photographs from different angles.

### Study Limitations

Our study had several limitations. First, the sample size was relatively small. Machine learning models perform better when trained using large-scale data sets. In addition, the generalizability of the prediction models needs to be validated in prospective multiple-center data sets. Second, this study was conducted in infants receiving IVI of conbercept, whereas reactivation rates may vary with different anti-VEGF agents and laser photocoagulation. Infants treated with other anti-VEGF agents and laser photocoagulation as the initial treatment for ROP should be included in future studies. Third, due to this study’s retrospective design, we could not obtain other potential predictive factors, such as posttreatment risk factors. Fourth, the retrospective design may limit the amount and quality of the collected data. A limitation of this model architecture is that it does not have an end-to-end design, which can lead to error accumulation. The errors in the output of each model may be amplified during the weighted averaging, affecting the final prediction results. One potential improvement could be to optimize the model through end-to-end joint training, using an ensemble learning framework or neural network to automatically adjust the weights and reduce error accumulation.

### Conclusion

In conclusion, we successfully developed and validated machine learning prediction models based on clinical risk factors and pretreatment retinal photographs for ROP reactivation. The promising results of the prediction models might aid in the early detection of ROP reactivation and decision-making processes in clinical practice.

## Appendix

Multimedia Appendix 1 Supplementary.

## Abbreviations

AdaBoost: Adaptive Boosting
AUC: area under the curve
CatBoost: Categorical Boosting
EMA: Exponential Moving Average
Grad-CAM: Gradient-Weighted Class Activation Mapping
IVI: intravitreal injection
LR: logistic regression
PMA: postmenstrual age
RF: random forest
ROC: receiver operating characteristic
ROP: retinopathy of prematurity
VEGF: vascular endothelial growth factor
XGBoost: Extreme Gradient Boosting

## Data Availability

The data sets are not publicly available because they are part of an ongoing study. However, the data are available from the corresponding author on reasonable request.
