# Supplementary material for: Prediction of Reactivation After Antivascular Endothelial Growth Factor Monotherapy for Retinopathy of Prematurity: Multimodal Machine Learning Model Study
Source: J Med Internet Res. 2025 Apr 23;27:e60367. doi: 10.2196/60367 (PMC12063557; doi:10.2196/60367)
Supplement: Multimedia Appendix 1 [file jmir_v27i1e60367_app1.docx]

Table S1. Algorithms’ parameters details of each algorithm.

| Algorithms | Parameters |
| --- | --- |
| AdaBoost | AdaBoostClassifier( learning_rate=0.01,  n_estimators= 1000,  estimator = DecisionTreeClassifier( class_weight={0:1,1:5} ,  max_depth=5,  max_features=4,  min_samples_leaf=20),  ) |
| RF | RandomForestClassifier(  max_depth=11,  n_estimators=700,  class_weight={0:1,1:3}  ) |
| XGBoost | XGBClassifier(  max_depth =7 ,  n_estimators = 1000 ,  scale_pos_weight = 2,  reg_alpha = 0,  reg_lambda = 0.1,  subsample = 0.9,  min_child_weight = 1  ) |
| CatBoost | CatBoostClassifier(  max_depth = 7 ,  iterations = 1000,  l2_leaf_reg = 6,  colsample_bylevel = 0.6,  subsample = 0.5,  class_weights = {0:1,1:4},  verbose=0  ) |
| Logistic Regression | LogisticRegression(class_weight={0:1,1:2},max_iter=1000,solver='newton-cg') |


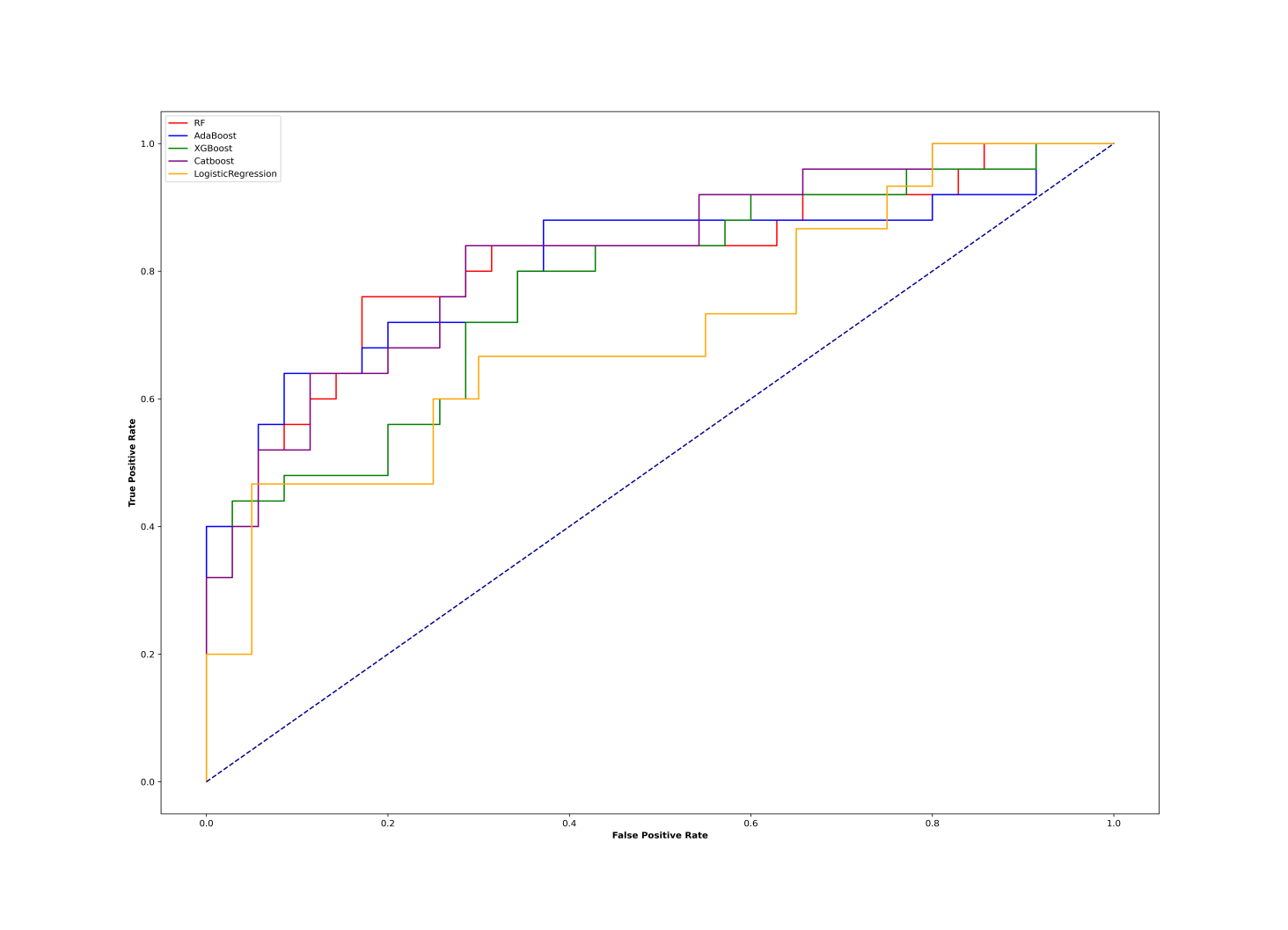


Figure S1. Receiver operating characteristic curves of machine learning predictive models for reactivation using all clinical factors. RF: random forest.


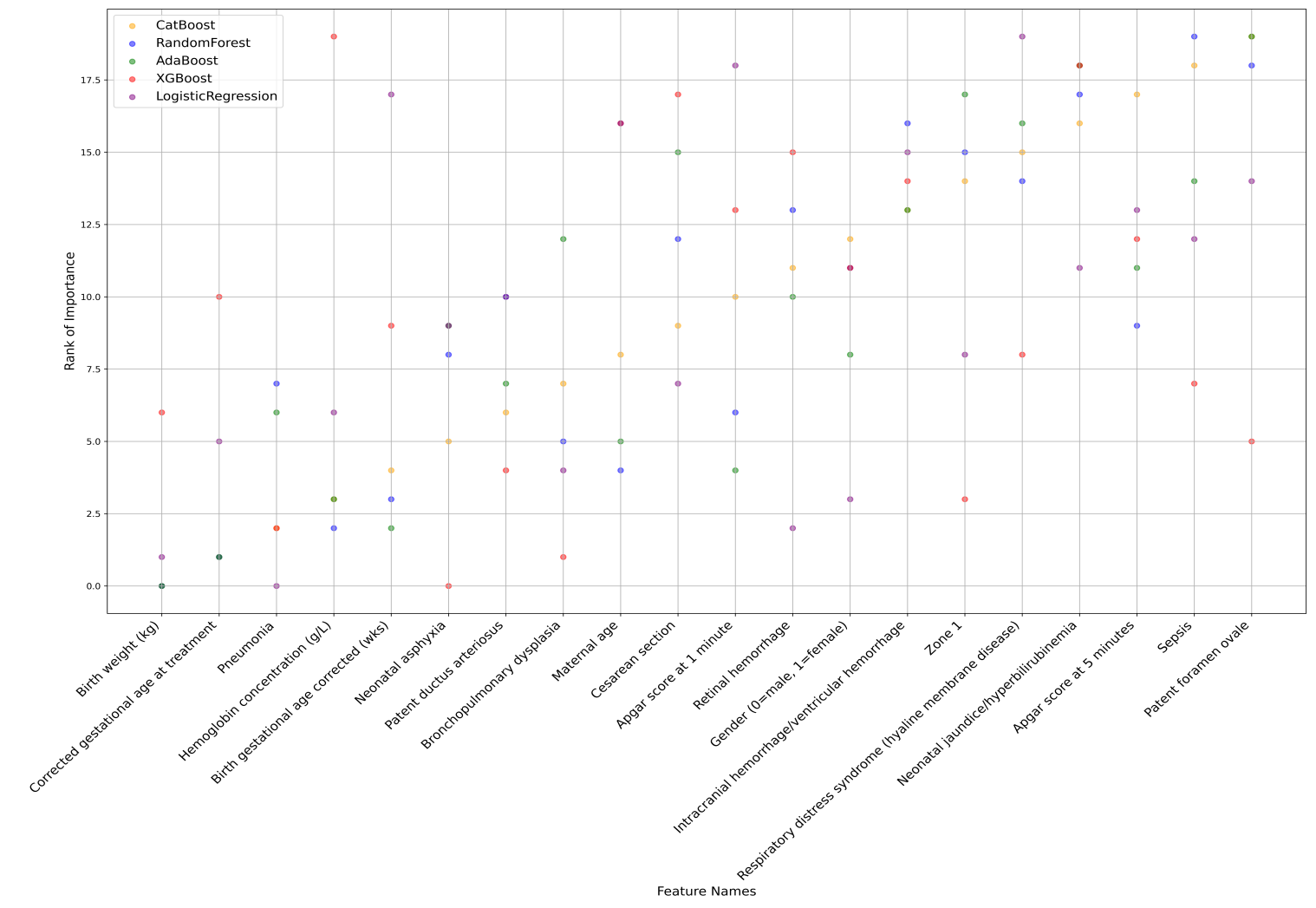


Figure S2. Classifier-specific predictive importance of each variable.


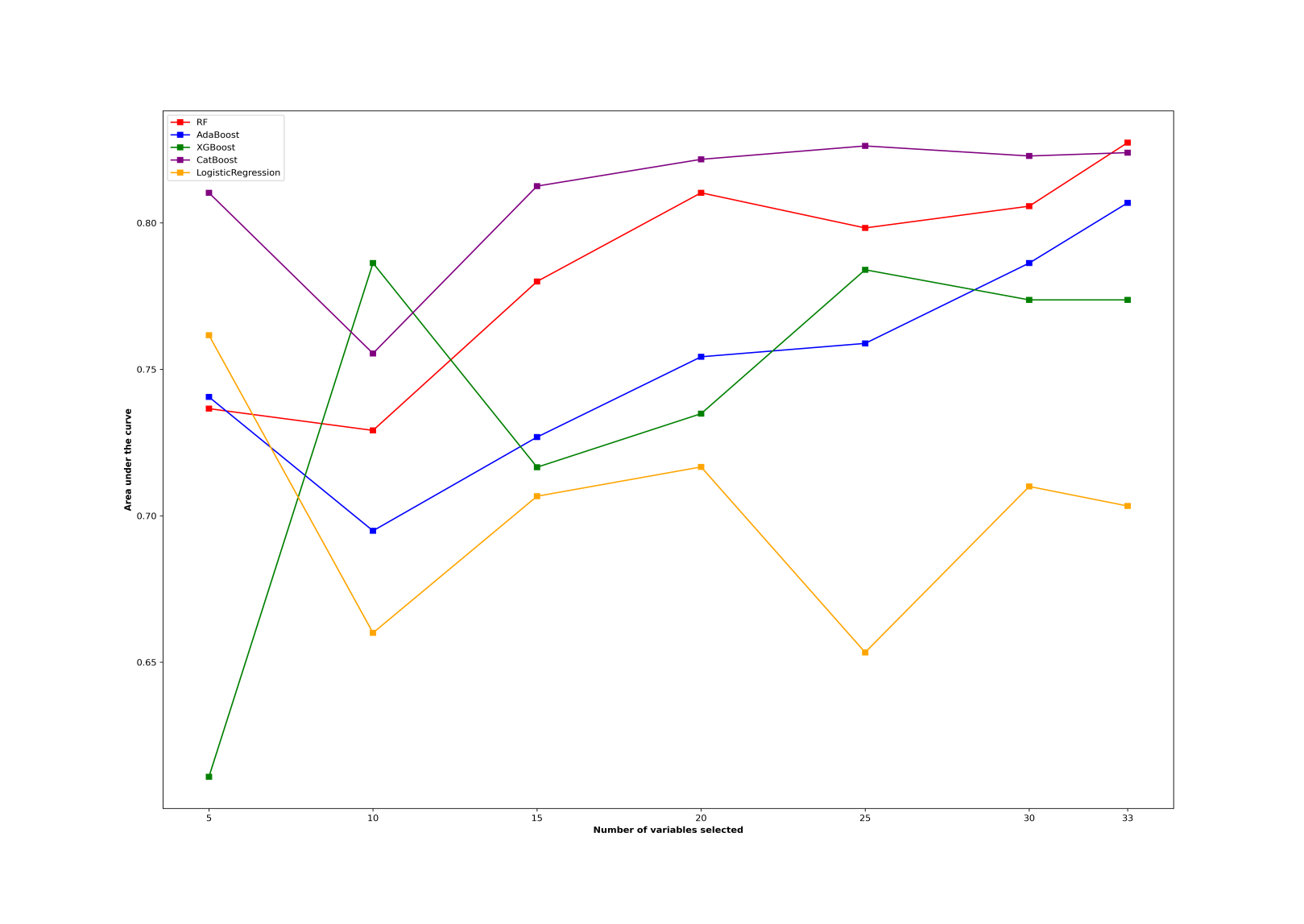
Figure S3. Receiver operating characteristic curves of

machine learning predictive models for reactivation on an increasing number of variables.


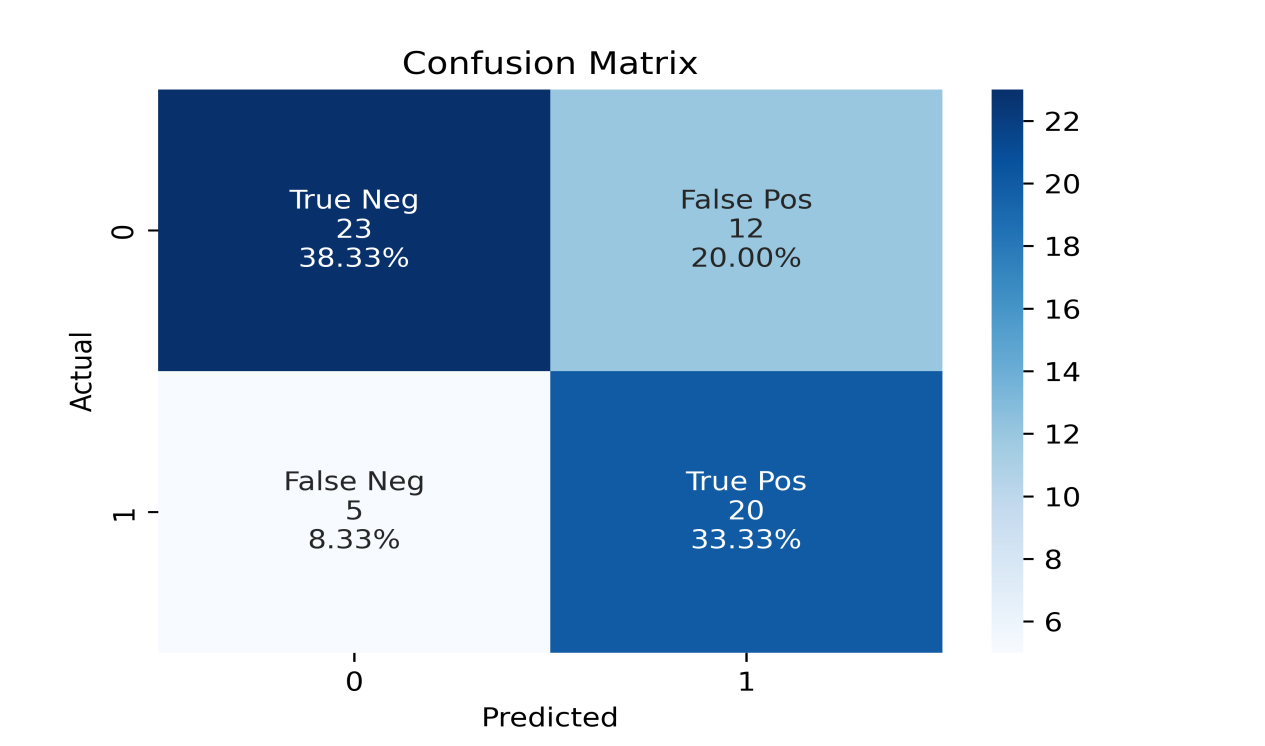


Figure S4. The confusion matrix of

predicting reactivation after anti-VEGF treatment for ROP using catboost in test cohort.


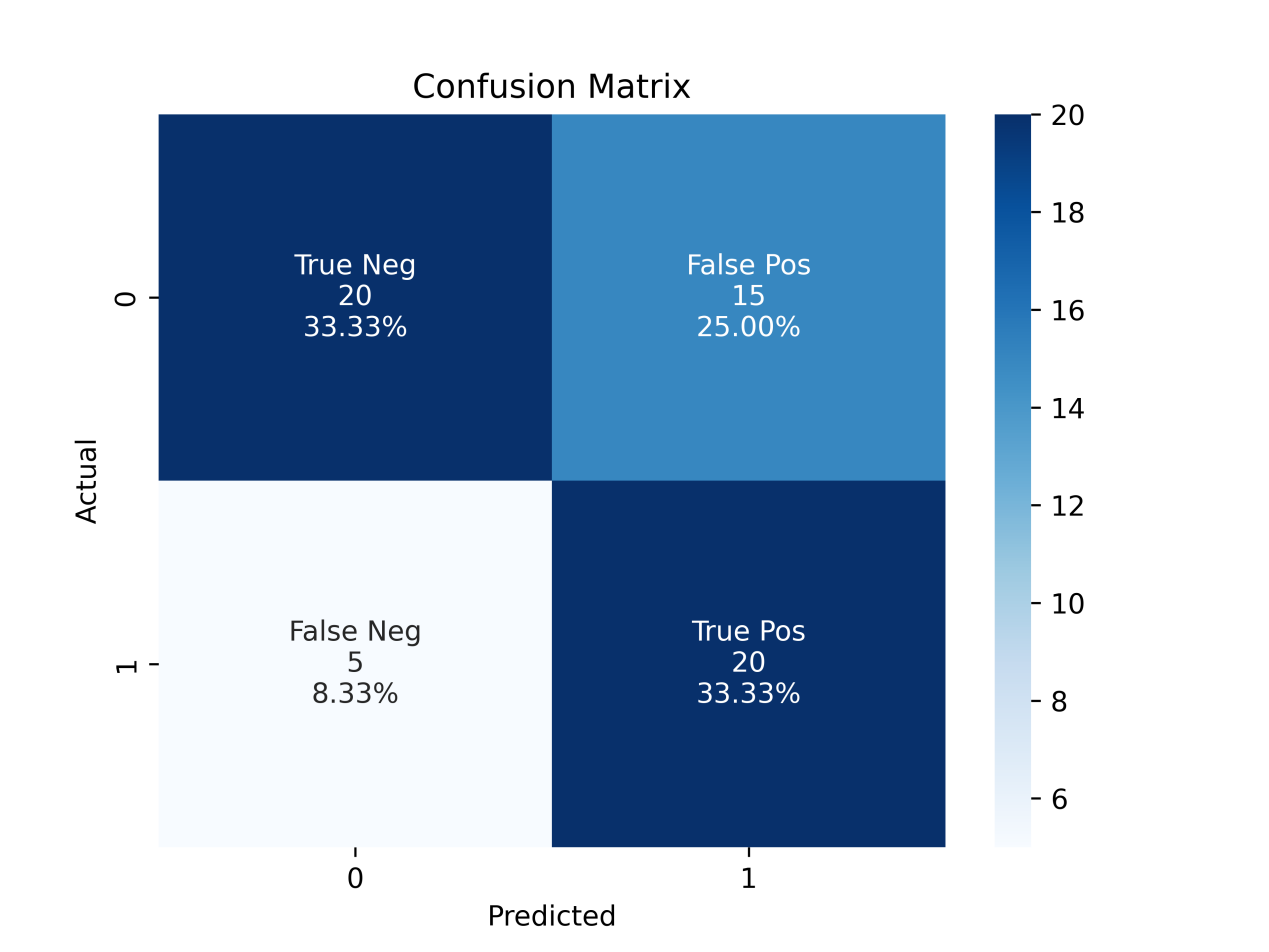


Figure S5. The confusion matrix of

predicting reactivation after anti-VEGF treatment for ROP using Resnet-50 in test cohort.


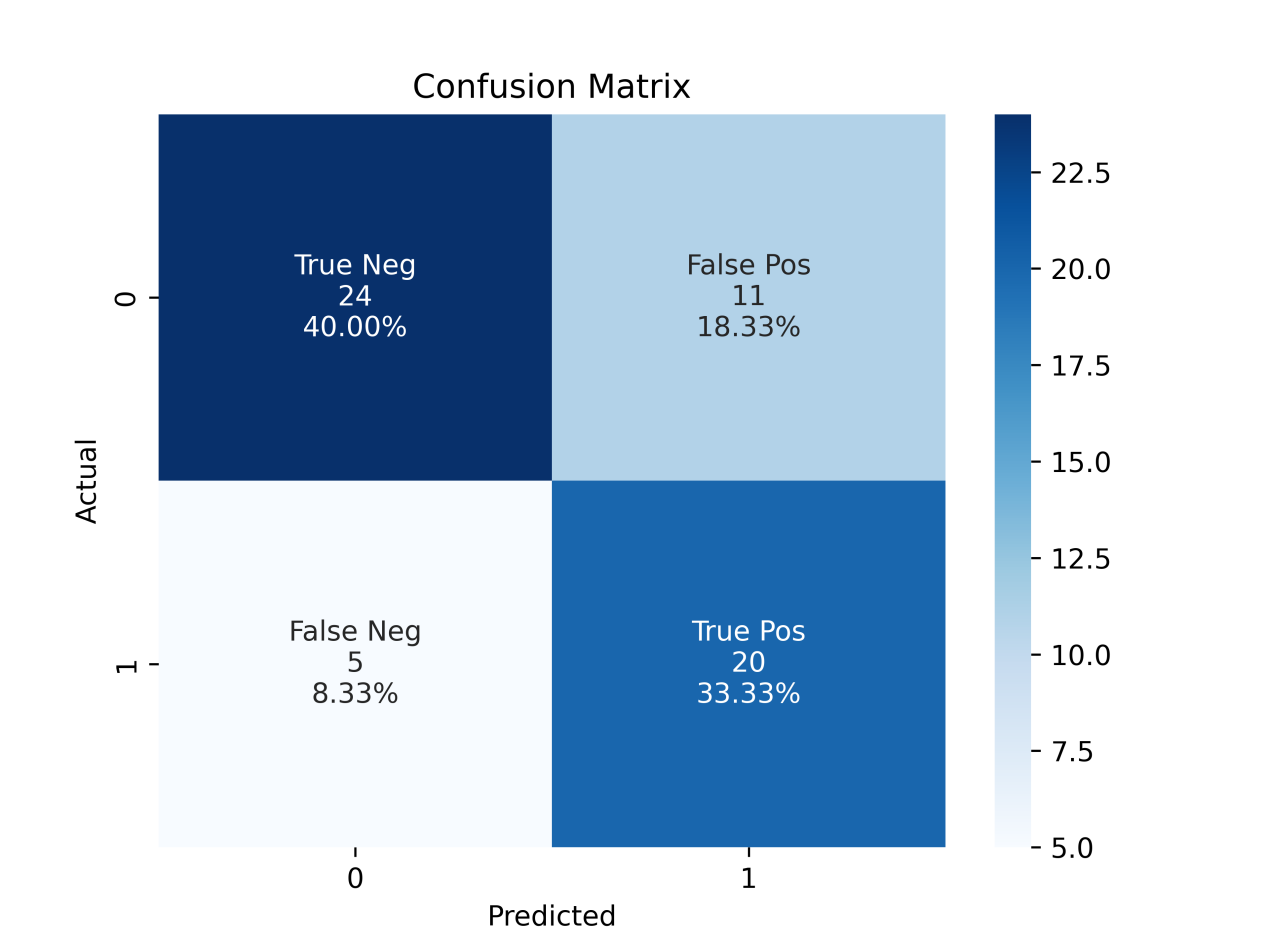


Figure S6. The confusion matrix of

predicting reactivation after anti-VEGF treatment for ROP in test cohort for fusion model.
